# Atomic Force Microscopy Nanomechanics of Hard Nanometer-Thick Films on Soft Substrates: Insights into Stretchable Conductors

**DOI:** 10.1021/acsanm.1c01590

**Published:** 2021-07-20

**Authors:** Giorgio Cortelli, Luca Patruno, Tobias Cramer, Mauro Murgia, Beatrice Fraboni, Stefano de Miranda

**Affiliations:** † Department of Civil, Chemical, Environmental and Materials Engineering, University of Bologna, Viale del Risorgimento 2, 40136 Bologna, Italy; ‡ Department of Physics and Astronomy, University of Bologna, Viale Berti Pichat 6/2, 40127 Bologna, Italy

**Keywords:** stretchable conductors, AFM nanoindentation, nanomechanics, FEM, hard on soft

## Abstract

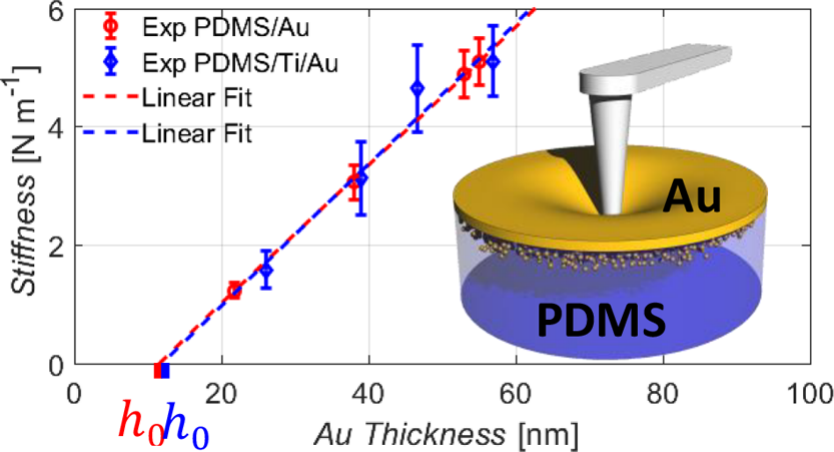

The nanomechanical properties of ultrathin and nanostructured films of rigid electronic materials on soft substrates are of crucial relevance to realize materials and devices for stretchable electronics. Of particular interest are bending deformations in buckled nanometer-thick films or patterned networks of rigid materials as they can be exploited to compensate for the missing tensile elasticity. Here, we perform atomic force microscopy indentation experiments and electrical measurements to characterize the nanomechanics of ultrathin gold films on a polydimethylsiloxane (PDMS) elastomer. The measured force-indentation data can be analyzed in terms of a simple analytical model describing a bending plate on a semi-infinite soft substrate. The resulting method enables us to quantify the local Young’s modulus of elasticity of the nanometer-thick film. Systematic variation of the gold layer thickness reveals the presence of a diffuse interface between the metal film and the elastomer substrate that does not contribute to the bending stiffness. The effect is associated with gold clusters that penetrate the silicone and are not directly connected to the ultrathin film. Only above a critical layer thickness, percolation of the metallic thin film happens, causing a linear increase in bending stiffness and electrical conductivity.

## 1 Introduction

Integration of advanced microelectronic sensor and actuator technology into devices with soft and stretchable mechanical properties is a major challenge for electronic materials science and device engineering.^1,2^ Low-invasive biomedical implants,^3,4^ soft robotics,^5^ or electro-mechanical energy harvesters,^6^ all rely on deformable electronic devices that are compliant to a mechanically demanding environment while maintaining their electronic functionality. Unfortunately, most of the currently known high-performance electronic materials are based on hard and stiff inorganic conductors and semiconductors. Only recent research demonstrated how structural engineering at the micro- and nanoscale permits to combine such hard materials with soft and elastic substrates, resulting in overall stretchable properties.^1^ The progress relies on the fact that hard inorganic materials can be bent with small forces, when patterned into ultrathin layers or nanowires.^7^ Such bending deformations can then be exploited to compensate tensile strain during device stretching. Examples of this approach are stretchable serpentine conductor lines,^8^ buckled conducting thin films,^9^ a kirigami,^10^ or island-based conducting network structures.^11,12^ So far, development and optimization of the stretchable surface patterns has been based on the analogy to their macroscopic counterparts and empirical findings. However, the nanoscale confinement of the thin inorganic layer and its adhesion to the elastic substrate can have a critical impact on the mechanical properties, changing the bending and fracture behavior. To take such effects into account and to achieve a rational optimization of wavy surface patterns for stretchable electronics, techniques and models are needed to characterize the nanomechanics of ultrathin hard layers on soft substrates.

Atomic force microscopy (AFM) has evolved in the last decade as a powerful tool to study the nanomechanics of complex surface structures. Advances in instrumentation have carried to a versatile tool, thanks to highly specified probes to investigate different mechanical properties at the nanoscale. Colloidal AFM probes have been widely used to measure the friction and adhesive surface properties of materials.^13,14^ The typical diameter of colloidal probes is in the order of a few micrometers to avoid penetration and fracture of the material under investigation. In a nanoindentation experiment, instead, the tip must locally penetrate the target surface; therefore, sharp AFM probes have been developed with a diameter in the order of 10 nm.^15^ The variation in the tip diameter allows operating in different force regimes with the same instrument. Important examples demonstrating the power of these techniques take into account the characterization of nanophase separated polymers or investigations of the cytoskeleton in living biological cells.^16^ Inspired by this success, first research groups have employed AFM to systematically investigate samples with more complex layered structures.^17^ Examples are thin layers on top of a substrate with softer or harder elastic properties, thin bilayers of polymeric materials with differing elastic moduli, or multilayered structures of layer-by-layer microcapsules.^13,18,19^ Indentation experiments on bilayer or multilayer structures face difficulties as analytical models are needed to extract the materials properties (i.e., elastic moduli and Poisson ratio) and structural information (i.e., layer thicknesses) from the measured force–indentation curves. For the case of homogeneous semi-infinite surfaces with isotropic elasticity, such models based on Hertz, Boussinesq, or Sneddon theories are available and are routinely applied in experiments.^16,20−22^ Instead for layered systems, no general analytical solution is available for the more complex nanoindentation process, and up to now, only few approximate or phenomenological models have been introduced. For example, under the assumption that the mechanical properties of the different layers do not differ widely, a perturbative approach has been developed to derive an effective, depth-dependent modulus that combines contributions from different layers and substrates.^18,19,23^ Other studies employ numerical finite element models to understand and quantitatively analyze indentation curves.^24^ Many studies on bilayers focus on the case of a soft top layer on a hard substrate as this is relevant to indentation studies on living cells where the so-called “bottom effect” introduces artifacts to the quantitative analysis of cell’s elastic properties.^25^ Inspiration for the derivation of analytical AFM nanomechanics models comes from classical mechanical engineering studies describing stiff, bendable layers on top of an elastic semi-infinite continuum to assess the stability of construction sites on soft soil.^26,27^ Advancing on this background, first analytical models have been derived in the literature to describe indentation of hard stiff nanometer-thick layers on soft substrates and open new opportunities to interpret experimental nanoindentation data.^28,29^

Here, we introduce AFM to investigate the nanomechanics of thin gold layers deposited on a silicone elastomer substrate. We consider such bilayers a model system for stretchable conductors based on ultrathin bendable metallic layers deposited on a soft substrate. Gold on silicone structures have recently been used to realize stretchable bioelectronic implants with low invasiveness due to their compliance to the mechanics of the surrounding tissue.^30,31^ The nanometer-thick gold film maintains its conductivity during stretching due to the formation of a microcrack pattern when a suitable layer thickness and adhesion promotor (titanium or chromium interlayer) are applied.^32,33^ With increasing strain, the nonconducting microcracks exposing the elastomer substrate open and the ultrathin gold layer takes the form of a network structure that compensates the tensile strain by bending deformations without breaking its interconnectivity.^34^ Therefore, the microcracked gold layer can maintain its electrical conductivity yielding a stretchable conductor. Gold layer thickness and adhesion are two fundamental parameters that decide on the performance of the stretchable conductor: too thin layers do not offer sufficient conductivity, whereas thick gold layers have a large bending stiffness and create only few but large fractures that interrupt conducting pathways during strain.

In order to understand the crucial role of thickness in the nanomechanics of such thin metallic films on elastic substrates, we introduce in this work indentation experiments combined with electrical characterizations. To interpret the indentation data, we exploit the analytical model provided by Lee^29^ and compare with finite element simulations. To our knowledge, it is the first time that the analytical model is confirmed experimentally and used to provide quantitative insight from AFM nanoindentation experiments. For the case of ultrathin metal films evaporated on a soft elastomer substrate, our results demonstrate the existence of a critical threshold of a few nanometers of thickness that decides on the mechanical properties. Below the threshold thickness, force–indentation curves show only a smaller increase in surface stiffness and maintain a Hertzian curved shape. Above the threshold, the metal film behaves as a bending plate and shows a linear force indentation curve that becomes independent of the shape of the identing tip. In this regime, the surface mechanics are fully dominated by the flexural rigidity of the metallic ultrathin film and the elastic modulus of the substrate. By performing measurements on gold layers with different thicknesses, we can relate the bending stiffness to the bulk elastic properties of the metallic thin film. Finally, comparison with the electrical conductivity of the gold-silicone layer shows that the threshold behavior in the indentation nanomechanics matches the onset of surface conductivity. Accordingly, we attribute the observed threshold to the onset of percolation of gold islands growing on the silicon substrate.

## 2 Experimental Section/Methods

### Polydimethylsiloxane (PDMS)/Au and PDMS/Ti/Au Preparation

PDMS is obtained by mixing a crosslinker and Sylgard 184 silicone in a ratio of 1:10. After intensive stirring, the mixture is put under vacuum so that the air bubbles are removed. The mixture is then poured onto a microscopy glass slide and spin-coated at 250 rpm for 3 min. Substrates are then stored for 1 h at 70 °C in an oven. Then, the titanium adhesion layer (1–2 nm thickness) and gold are deposited on the glass/PDMS substrates by thermal evaporation (source sample distance = 25 cm; vacuum pressure = 5.5 10^–6^ mbar).

### AFM Characterization

**Figure 1:**
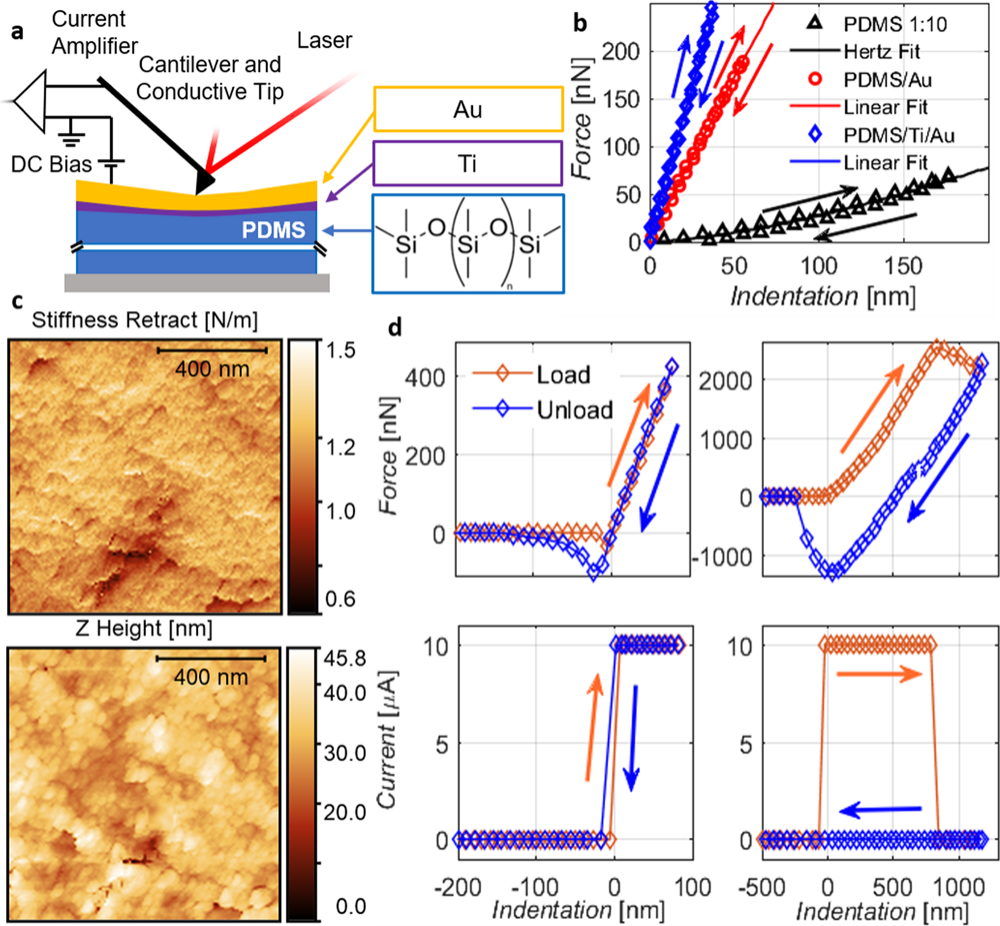
Nanomechanical and electrical characterization of thin gold films on the elastomeric substrate. (a) Scheme of the AFM experimental setup. (b) Force–indentation curves on thin metal films of thickness 38 nm (Au, red) and 57 nm (Ti/Au, blue), compared to the pure PDMS substrate (black). (c) Height and stiffness map (128 × 128 pixel) of Au deposited on PDMS. (d) Force–indentation and conductive AFM curves of PDMS/Ti/Au (40 nm thickness) with different force limits.

A Park System’s NX10 AFM was used in the experiments. A Rocky Mountain Nanotechnology probe 25Pt300B was used to perform simultaneously conductive AFM and force spectroscopy. For normal indentation experiments, we used nonconductive tips such as Mikromash’s NSC36B and NCHR from Nanosensors. To study the linear elastic regime, we typically used a setpoint of 400 nN and an indentation speed of 3 μm s^–1^. The range of Z height scanned is defined to obtain at least a 200 nm baseline before the contact between the tip and the sample takes place. Prior to each experiment, the tip sensitivity and force constant are calibrated by indentation on a silicon surface and thermal tune method. The uncertainties reported for *h*_0_^mec^ and *E*_Au_ are obtained by error propagation of the uncertainties estimated by the linear fit of the stiffness-thickness data and the relative error of the spring constant of the AFM cantilever, found during calibration to be approximately 5%. The AFM tips 25Pt300B, NSC36B, and NCHR have a spring constant equal to 18, 11, and 5 N m^–1^, respectively. The stiffness map shown in Figure 1c was obtained by the PinPoint mode of the AFM. NCHR tips in noncontact mode were used to measure the gold film thickness. To do so, during the gold deposition, we placed a glass carrier near each PDMS sample, and we covered partially the glass. The thickness was measured by scanning 40 μm^2^ area between the covered and uncovered part of glass/Au samples.

To investigate the dependence of the force–indentation curves on the tip radius, we vary the tip curvature by applying frictional wear. We first characterize the tip radius by performing NCM measurements on a tip-shape characterization sample (MicroMasch, PA01). We then approach the AFM tip on a microscopy slide glass surface and scratch in a controlled way (200 nm, 10 lines, 50nN Setpoint). Finally, the new radius is estimated as fitting parameters of force–indentation curves of pure PDMS with a known elastic modulus, according to the Hertz model.

### Van der Pauw Measurements

A current of 100 mA is injected into the samples by means of a Keysight SMU. Copper tape and silver paste are used to favor electrical contact between the gold thin film and the SMU. We measure the resistance of the film using four different configurations by varying the side in which the current is injected. The voltage is measured on the opposite side as in the standard van der Pauw setup.^35,36^

## 3 Results

Figure 1a shows the basic experimental configuration that we exploit to investigate the nanomechanics of thin gold films on elastomeric substrates. Investigated samples contain a 0.7 mm thick PDMS layer attached to a glass carrier. On top of the PDMS films, we thermally evaporated a thin titanium adhesion layer followed by a thicker gold layer (see the Section 2 for details). In an atomic force spectroscopy experiment, we push the tip against the surface and measure how the force increases as a function of the sample indentation. Typical indentation depth is about 100 nm. The thickness of the PDMS substrate ensures that the presence of the glass carrier is negligible as we probe less than 1% of the sample. Typical curves obtained for hard thin films on soft substrates such as PDMS/Ti/Au (blue) and PDMS/Au (red) are shown in Figure 1b. The curves show an immediate linear increase in force with indentation. Also, we observe an elastic response in which there is no hysteresis between the loading and unloading curves. This finding is at strong difference to indentation experiments performed on the pure PDMS substrate (black line). For PDMS, forces are significantly lower and show a super-linear increase with indentation, precisely following the prediction of the Hertz model.^16,20−22^ By performing indentation experiments at different positions on the surface, we can verify that the increase in stiffness due to the thin metal layer is constant throughout the surface (Figure 1c). From the high-resolution stiffness map shown in Figure 1c, we obtain an average value of 1.3 N m^–1^ for a 38 nm Au film. The small standard deviation of 0.07 N m^–1^ points to a homogeneously deposited metal film. Little variations only emerge due to local fluctuations in film thickness and grain structure as seen in the height map (Figure 1c). Differently, measurements at the border between stiff Au islands and zones with exposed PDMS show an abrupt decrease of stiffness (Figure S1a).

To understand possible perforation of the metal film during indentation, we increase the maximum indentation force and combine the measurement with conducting AFM. In this way, it is possible to study the mechanical response of the bilayer while monitoring the current flowing from the film into the tip. Figure 1d shows such a measurement with a force limit of 400 nN. Within this range, the response is linear and reversible, and a constant current is monitored between the tip and sample. Increasing the force limit (Figure 1d right), we enter a second regime in which at a critical force value (here 2000 nN), the current is interrupted, and indentation continues without further need to increase the force. During unloading, a strong hysteresis emerges in the indentation curve and the current remains at zero. These findings point to a fracture of the surface at critical forces. This is also confirmed by the surface topography measured after the indentation that shows a clear tip imprint once the threshold force is exceeded during indentation (Figure S1b).

### 3.1 Analytical Model

**Figure 2:**
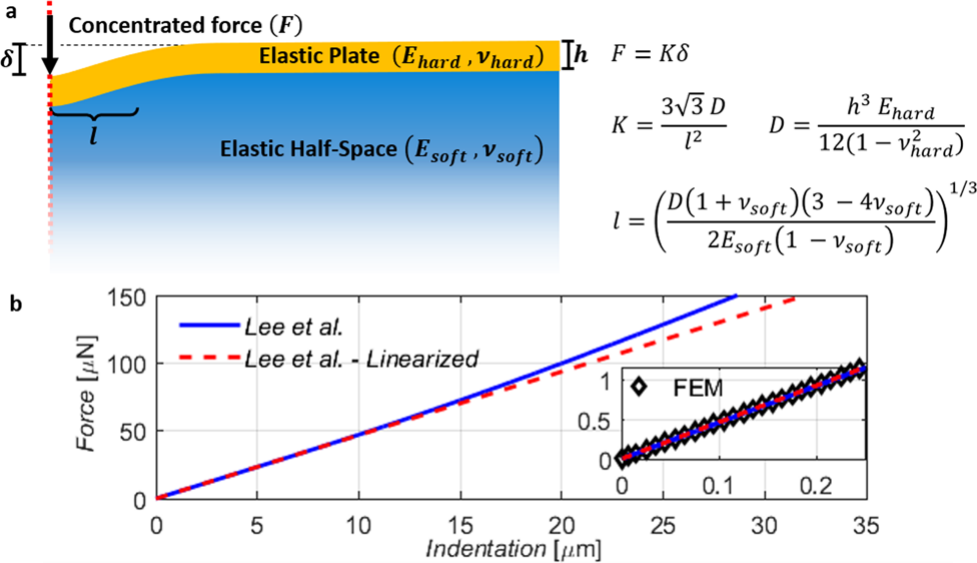
Indentation models for hard films on soft substrates: (a) Scheme showing the main parameters. On the right, the main equations of the model in the case of indentation of a uniform layer bonded to an elastic half-space are reported. (b) Calculated force–indentation curves according to the analytical solution (blue) and its linearized version (red).^29^ The two curves start to diverge significantly at indentations exceeding 10 μm. The inset shows the calculated indentation curves in the experimental range of indentation. The black data correspond to the result of the FE numerical simulations.

From the experiments described above, profound differences emerge between the response of the pure PDMS and that of layered samples such as PDMS/Ti/Au and PDMS/Au: the presence of gold not only stiffens the system but also modifies the force–indentation curve, which no longer follows the Hertz model. To obtain a quantitative description of this behavior, we consider the analytical model proposed by Lee et al.,^29^ which represents the layered system as a thin elastic plate bonded to an elastic half-space, see Figure 2a. As described in the Supporting Information, the model yields a nonlinear analytical solution to the indentation mechanics. For our case, we further simplify the model by representing the indenter as a concentrated force *F* and neglecting local Hertz-like deformations in the stiff metal layer. These assumptions are justified by the large mismatch of the elastic modulus of the metal layer with respect to the substrate. The resulting linear relation between force and indentation *F* = *K*δ is characterized by the stiffness *K*, i.e., the slope of the force–indentation curve, that can be expressed as follows (eq 1):
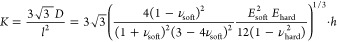
1where *E*_hard_, *E*_soft_, *ν_hard_*, and *ν*_soft_ are the elastic moduli and the Poisson’s ratios of the hard thin layer and of the soft substrate, respectively, and *h* is the thickness of the thin layer. We note that the linearization of the force–indentation curves removes any functional dependence on the indenter geometry. This differs greatly from other indentation models proposed in the literature such as the Hertz model or more advanced bilayer models.^18,20−22,37^ In fact, in such models, the shape of the indenter and local effects under the tip play a crucial role, as they determine the effective contact area, which is a function of the indentation depth. For the here investigated case of *E*_hard_ > > *E*_soft_, the linearized model appears suitable to represent the hard on soft bilayer behavior at small indentation. This is justified by considering the characteristic length scale *l* defining the radius of the area on the rigid bending plate below the indenter, which undergoes relevant vertical displacement. The characteristic length can be written as follows (eq 2):
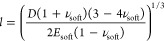
2where *D* = *h*^3^*E*_hard_/12(1 – *ν*_hard_^2^) is the flexural rigidity of the plate.^29^ Using the typical experimental parameters, a value of *l* = 1.1 μm is expected and is clearly much larger than typical contact areas between the indenting tip and the surface. Accordingly, the indenter can be modeled as a concentrated force applied to the plate. Furthermore, the model neglects the shear deformability of the plate,^26^ which requires 

The conditions for the linearized solution are met when the ratio of the elastic moduli of the thin film and the substrate is high enough. In our case, the ratio is in the order of 10^4^. Figure 2b compares the analytical solution and its linearized version. For the computations, we assumed bulk values for PDMS and Au as found in the literature (*E*_hard_ = 78 GPa, *E*_soft_ = 1.55 MPa, *ν*_hard_ = 0.44, and *ν*_soft_ = 0.5), a gold layer thickness of *h* = 44 nm, and a spherical indenter of radius *R* = 20 nm. As it is clearly visible from the plot, the linear term is dominant up to approximately 10 μm indentation. However, experimentally, a typical maximum indentation to perform AFM force spectroscopy is around 250 nm (Figure 2b inset). Furthermore, the fracture of the gold film occurs at loads in which the two curves are still overlapped. For example, with a tip of radius around 20 nm, the fracture occurs at around 500 nN of load applied.

To further confirm the validity of the aforementioned conditions, we perform numerical simulations based on the finite element method. The input parameters of the numerical simulations are set equal to the ones reported for the comparison between the analytical solution and its linearized version. The inset in Figure 2b shows the numerical results in the typical experimental operating range compared to the two analytical curves. More details about numerical simulations can be found in the Supporting Information.

### 3.2 Experimental Validation of the Model

**Figure 3:**
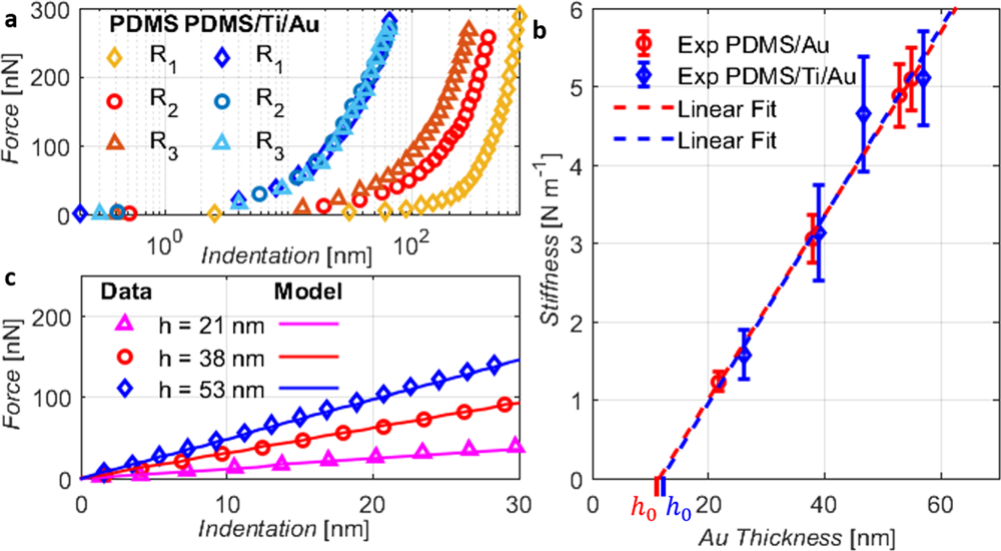
Experimental validation of the indentation model: (a) Force–indentation curves acquired with three different AFM tips on both pure PDMS (warm colors) and PDMS/Ti/Au (blue colors). (b) Stiffness dependence on the thin film thickness. Note that the experimental data shown in Figure 3b correspond to the average of the stiffness of the loading and unloading curves. This avoids possible systematical errors due to thermal drift during the AFM acquisition. (c) Comparison between the experimental indentation curves and the model predictions for PDMS/Ti/Au samples of different thicknesses (*h* = 21 nm, *h* = 38 nm, and *h* = 53 nm).

To validate the model, we first investigate the impact of the indenter geometry by means of force spectroscopy with three different AFM tips, characterized by increasing radii of curvature, called *R*_1_ (20 nm), *R*_2_ (135 nm), and *R*_3_ (490 nm), respectively. The acquired curves are shown in Figure 3a in different shades of blue. We compare with indentation experiments performed on pure PDMS as represented in warm colors. For PDMS, as expected from the Hertz model, a strong dependence on the tip radius is found. Differently, the acquisitions made on PDMS/Ti/Au (cold colors) show superimposed force–indentation curves. Therefore, the experiments confirm the absence of any impact of the tip geometry, as predicted by eq 1. Accordingly, the representation of the AFM tip as a concentrated force in the analytical model appears to be well justified.

Next, we investigate the variation of the response in samples with different gold thicknesses (20–60 nm). A linear relationship between thickness and stiffness, as predicted by the analytical model (eq 1), is confirmed for both PDMS/Ti/Au and PDMS/Au, as shown in Figure 3b, where also the linear fit of the data is shown. We notice that the linear relation reported in eq 1 passes through the origin. Thus, the stiffness vanishes as the gold thickness goes to zero. Instead, Figure 3b shows that the linear fit of the experimental data results in an offset different to zero and extrapolation suggests a nominal gold layer thickness of *h*_0_^mec^ at which the stiffness is zero. On this regard, we note that the nominal thickness of the evaporated metal film is evaluated by a quartz crystal balance calibrated by AFM measurements of layers deposited on a glass support. The presence of *h*_0_^mec^ in the experimental data indicates the existence of an ineffective thickness of the metal film, which does not contribute to the stiffness of the metal layer. Taking such effect into account, eq 1 can be rewritten as follows:
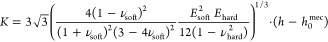
3

In our case, in the absence of titanium (red curves in Figure 3b), the evaluated ineffective thickness value is *h*_0_^mec^ = 11 ± 2 nm, while, for PDMS/Ti/Au (blue curves in Figure 3b), we obtain *h*_0_^mec^ = 12 ± 3 nm. We estimate *E*_hard_ from the slope of the linear fit of the thickness-stiffness experimental data using eq 3. The Poisson’s ratio of the hard film is set equal to its bulk counterpart, *ν*_hard_ = 0.44. With the above settings, eq 3 yields *E*_hard_ = 103 ± 21 GPa for PDMS/Au. No significant impact of the titanium adhesion layer on *E*_hard_ is observed, as it is estimated to be *E*_hard_ = 110 ± 22 GPa for PDMS/Ti/Au (Figure 3b blue data points). Once the relation between film thickness and stiffness is found, the linear force–indentation curve can be obtained. Figure 3c shows a comparison between the model predictions and experimental data, showing a good agreement between the two.

### 3.3 Nanomechanical and Electrical Properties Influenced by Thin Film Growth

**Figure 4:**
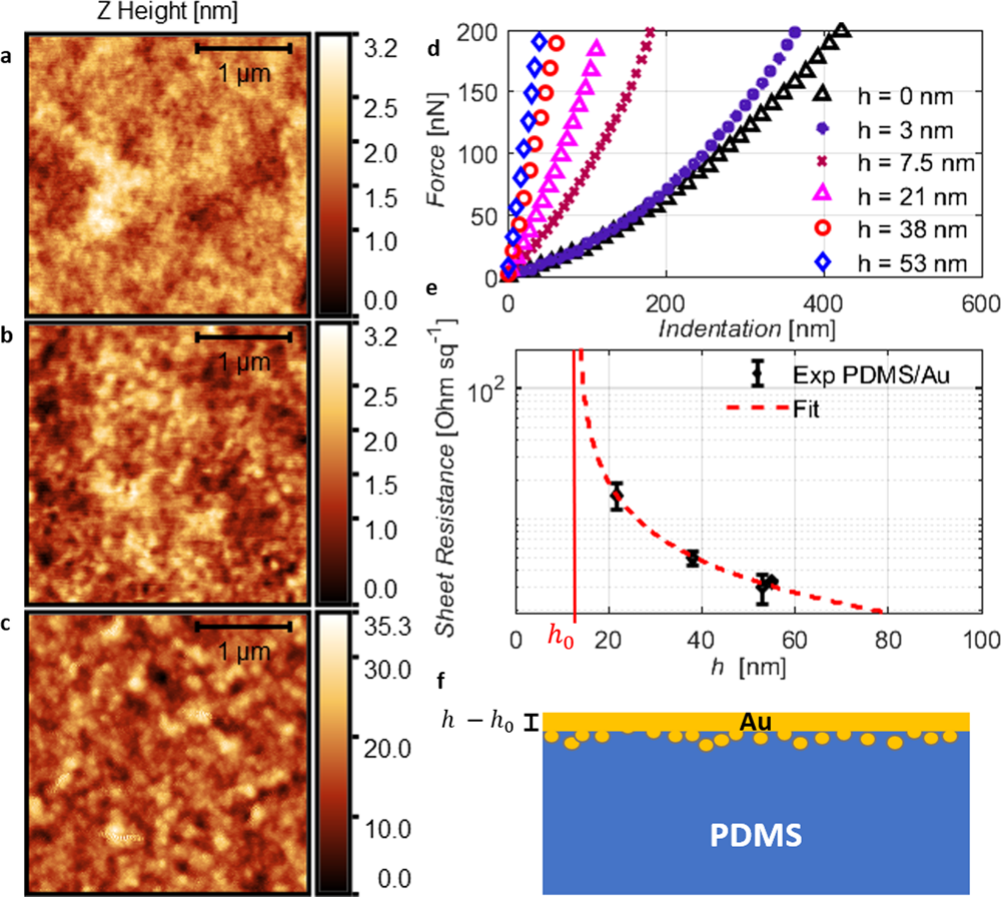
Relationship between gold layer thickness and electrical properties: (a-c) Noncontact mode AFM images acquired on (a) pure PDMS and PDMS/Au with gold film thickness (b) below and (c) above the threshold value *h*_0_^mec^. (d) Force–indentation curves of PDMS/Au with gold film thickness above and below the threshold value *h*_0_^mec^. (e) Sheet resistance of samples with different film thicknesses. (f) Qualitative representation of the effective thickness interpretation.

In the former paragraph, we found that the nominal thickness of the hard thin film systematically leads to an overestimation of the bilayer stiffness. Then, the presence of a mechanically ineffective layer of thickness *h*_0_^mec^ has been introduced to explain the mismatch, leading to a good approximation of the experimental data. To understand the reasons for the presence of such a mechanically ineffective part of the gold layer, we perform AFM analysis of several samples with thicknesses lower than *h*_0_^mec^. Figure 4a,c, compares the surface morphologies of the pure PDMS substrate and two bilayer samples with gold thicknesses 7.5 and 53 nm. The deposition of the 7.5 nm Au layer did not increase the overall surface roughness, which is characterized by a standard deviation of the height equal to 0.31 nm, similar to the results obtained for pure PDMS (std = 0.36 nm). The only difference is that the small variations in surface height show a more granular structure.

Considering now the morphology of a film with thickness greater than *h*_0_^mec^, a completely different picture emerges: the deposition of a larger amount of gold leads to a rough surface (std = 4 nm) characterized by clusters whose diameter measures several nanometers. These images indicate that a continuous gold film is formed only for thicknesses greater than the threshold thickness *h*_0_^mec^. Below such a thickness, the deposited gold clusters seem to permeate into the elastic polymer substrate giving rise to a diffused interface. Consequently, the deposited gold clusters do not connect between each other and offer no resistance against bending of the surface. This picture is further supported by the force–indentation curves acquired for the samples shown in Figure 4d. For samples with *h* < *h*_0_^mec^, the mechanical response follows the Hertzian curve.

The presence of a diffused interface is further confirmed by additional independent measurements of the electrical sheet resistance of the deposited films. Below *h*_0_^mec^, we do not find any conductive behavior of the gold film. For thicker layers, above the onset of percolation, electrical conductivity is established. Using a van der Pauw contact geometry, the sheet resistance, *R*_s_^Au^, of the thin film is experimentally measured.^35,36^ By using simple geometric arguments, we express the thickness dependence of the sheet resistance as follows:
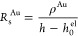
4where ρ^Au^ is the resistivity of gold, *h* is its nominal thickness, and, in analogy to the proposed mechanical model, an ineffective thickness, *h*_0_^el^, has been introduced. Below such a threshold, the gold film is not interconnected and is not conductive. We note that a similar threshold thickness for electrical conductivity was also reported by Graudejus et al. for thermally evaporated metallic thin films on silicone substrates.^38^

In analogy to the procedure used to study the mechanical behavior, it is possible to estimate *h*_0_^el^ and ρ^Au^ by fitting the experimental data according to eq 4. As the sheet resistances differ by orders of magnitude, we fit the measurement data on a logarithmic scale as shown in Figure 4e. In this way, we evaluate *h*_0_^el^ = 13 ± 3 nm for PDMS/Au samples, which compares well to the mechanical measurements. The estimated resistivity ρ^Au^ = 127 ± 21 Ω nm is significantly larger than its bulk equivalent. The reason for the larger resistivity is associated with the disordered, cracked microstructure of the thin metallic film.

Both mechanical and electrical measurements point to a critical thickness *h*_0_, below which the metal layer is not continuous. A simplified scheme of such a situation is depicted in Figure 4f. Such diffused interfaces have been described for several cases in which metals were thermally deposited onto organic or polymeric substrates.^16^ Migration of metal clusters into the polymer is possible due to the high kinetic energy and the small size of the arriving clusters. With the ongoing deposition, larger clusters start to condense closer to the interface and increase in size, so that penetration into the polymer becomes more and more unlikely. Finally, when the threshold thickness is reached, clusters percolate and a continuous film starts to build up. The amount of material diffused into the polymer contributes neither to the mechanical bending stiffness nor to the electrical conductivity.

## 4 Discussion and Conclusions

Our work demonstrates how AFM indentation experiments can be performed and interpreted to investigate the nanomechanics of hard nanometer-thick metallic films on soft elastomer substrates. By combining the mechanical nanoindentation experiments with conductive AFM, we were able to distinguish perforation of the conductive surface that leads to irreversible damage and conductance failure. Instead, when nanoindentation experiments are performed with forces remaining below the critical fracture force, the response is elastic and reversible. In this elastic regime of indentation, we find that the nanomechanics become independent of the indenter geometry and scale linearly with layer thickness. We achieve a quantitative description of this behavior by referring to the analytical model proposed by Lee et al. for the indentation of a hard uniform layer bonded to a soft, elastic half-space.^29^ We show that a linearized version of the model already accounts for all the observed phenomena in the experimental parameter range and allows one to relate the observations to mechanical material properties such as the elastic modulus and Poisson ratio of the involved materials. By comparing also to finite element simulations, we find that the linearized model holds due to the large mismatch in elastic moduli between the soft substrate and hard metallic thin film (). For such a situation, the metallic film behaves effectively as a bending plate for which it is possible to disregard local deformation under the tip.

For the investigated case of gold thermally deposited on the silicone elastomer, we exemplify how the described method enables a deeper understanding on the relation between the nanometer-thick film structure and its nanomechanical properties. By varying the gold layer thickness, we demonstrate that a significant increase in layer stiffness only happens above a threshold thickness *h*_0_^mec^. We explain the presence of this threshold by the penetration of initial evaporated gold clusters into the polymer substrate to create a diffuse interface that retards percolation of interfacial clusters into a continuous film. The existence of a percolation threshold is also confirmed by electrical measurements of surface conductivity that show an onset at a similar layer thickness. Once percolation is achieved, the film builds up a flexural rigidity and significant surface stiffening happens. From the quantitative analysis of the surface stiffness, we find a local Young’s modulus of such ultrathin gold films of 103 ± 21 GPa. The value matches within its uncertainties the gold elastic modulus obtained in macroscopic deformation experiments. Interestingly, we find no significant impact on the investigated thin-film nanomechanics by the thin titanium adhesion layer used to bind gold stronger to the elastomer substrate and to prevent delamination. This observation agrees with the analytical model presented by Lee et al.^29^ in which no effect of the adhesion between the layer and substrate is found if the latter is assumed incompressible (*ν*_soft_ = 0.5). We further note that in our experiments, delamination effects are not relevant, but they become important once a microcracked network is established to achieve reversible stretching stability.

In conclusion, our work demonstrates a quantitative analysis of the surface nanomechanics of thin hard metallic films on soft substrates. It enables us to extract the effective layer thickness and the local Young’s modulus of the thin metallic layer. Both are parameters that are crucial to understand and predict the properties of such films once they are employed as stretchable conductors where their bending is needed to compensate tensile strain. So far, nominal thickness values and bulk elastic moduli were used as parameters when modeling such systems to optimize strain compensating geometries.^24^ Instead, our method provides an experimental access to the relevant parameters, thereby paving the way toward a quantitative understanding and optimization of hard on soft interfaces for stretchable electronics.

## Supporting Information Available

Additional AFM images, introduction of the analytical indentation model and linearization, and FEM numerical simulation (PDF)
