# Supplementary material for: Atomic Force Microscopy Nanomechanics of Hard Nanometer-Thick Films on Soft Substrates: Insights into Stretchable Conductors
Source: ACS Appl Nano Mater. 2021 Jul 20;4(8):8376–82. doi: 10.1021/acsanm.1c01590 (PMC8411650; doi:10.1021/acsanm.1c01590)
Supplement: Supplementary file 1 — an1c01590_si_001.pdf [file an1c01590_si_001.pdf]

Supporting Information

Atomic Force Microscopy Nanomechanics of Hard Nanometer-Thick Films on Soft Substrates: Insights for Stretchable Conductors

Giorgio Cortelli [a], Luca Patruno [a], Tobias Cramer [b], Mauro Murgia [b], Beatrice Fraboni [b], Stefano de Miranda* [a]*

a) Department of Civil, Chemical, Environmental and Materials Engineering, University of Bologna, Viale del Risorgimento 2, 40136, Bologna, Italy

b) Department of Physics and Astronomy, University of Bologna, Viale Berti Pichat 6/2, 40127, Bologna, Italy

E-mail: tobias.cramer@unibo.it , stefano.demiranda@unibo.it

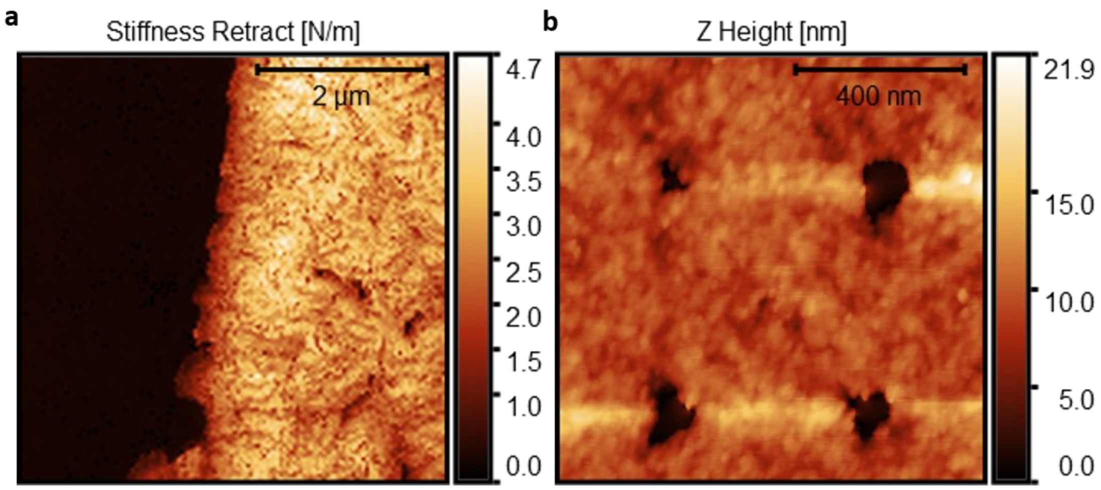

Figure S1. a) Stiffness map (128 x 128 pixel) on region of PDMS/Au bordering to pure PDMS region. b) Tip imprints left on the gold film by reaching the second regime during nanoindentation.

1. Introduction of the analytical indentation model and linearization

The analytical model for the indentation of a uniform stiff layer bonded to a homogeneous half-space has been proposed by Lee *et al.*[29] The relation between the force (***F***) and the indentation (***δ***), can be written as:

$$\delta = \frac{F}{D}\left\{\frac{l^2}{3\sqrt{3}} - \frac{l^2}{2\pi}\left[1 + \frac{\pi D}{FR}\right]\exp\left(-2C - \frac{2\pi D}{FR}\right) + \frac{1}{4h^2}\left(\frac{D}{4FR}\right)^{\frac{1}{3}}\right\} \tag{S1}$$

where C is Euler's constant, R is the radius of the rigid spherical indenter, h is the plate thickness, D is the flexural rigidity of the plate and l, reported in **Equation 2**, is a characteristic length of the problem. Notice that Equation 2 is valid if no sliding is allowed between the plate and the substrate. When sliding is allowed a different expression for l is provided in [29]. However, differences are limited to the 10% and the two coincide for incompressible substrates ($\nu_{soft} = 0.5$). In **Equation S1** the first term gives the contribution of a concentrated force representative of the indenter. The second term is a correction that takes into account the distribution of the load along the ring of contact between the indenter and the plate. The third term considers Hertz-like local deformations of the plate under the indenter. It must be highlighted that Equation S1 has only one linear term and that all the other terms depend on the indenter radius. Therefore, assuming the load to be concentrated leads to the linear force-indentation relation:

$$\delta = \frac{F}{K} \tag{S2}$$

where K is the stiffness of the bilayer given in Equation 1.

2. FEM Model

We investigate also with numerical simulations based on FEM the independence of the force-indentation curve on the indenter radius. **Figure S2a** shows the setup of the axisymmetric FE model, together with a detail of the mesh under the tip. A mesh entirely composed of quadrilateral elements is adopted. The hard thin layer and the soft substrate are assumed as

homogeneous, isotropic, linear elastic materials. We set the input parameters of the numerical simulations equal to the values used to compare the analytical solution and its linearized version (see Figure 2b). The indenter is assumed to be a spherical rigid body. Three radii have been considered, namely 20, 100, 400 nm. The computational domain is a square of side equal to 50 μm. The nodes on the axis of symmetry are fixed in the horizontal direction, while those at the bottom are constrained in the vertical direction. Concerning the numerical treatment of the contact problem, the indenter is defined as a master surface, while the layer is defined as a slave surface, together forming a contact pair. The normal contact is set to avoid interpenetration of the two bodies, while the tangential behavior is assumed to be frictionless. The hard film is assumed to be connected to the substrate without the possibility to slide.

It must be noticed that an infinite half-space is considered in the analytical model. Therefore, when comparing results of the analytical model to FEM results it is essential to ensure that the numerical domain is big enough with respect to l. For example, a variation of the domain side from $7l$ to $50l$ leads to force estimations that differ of 2%.

Figure S2b reports the output of three numerical simulations performed varying the indenter radius. It must be highlighted that we measure the displacement at the interface between the rigid indenter and the hard layer. In this way, also local deformations under the indenter are taken into account. Nevertheless, the three simulated force-indentation curves are practically coincident. Therefore, also the FEM numerical simulations confirm the suitability of the linearized version of the analytical model for the considered application.

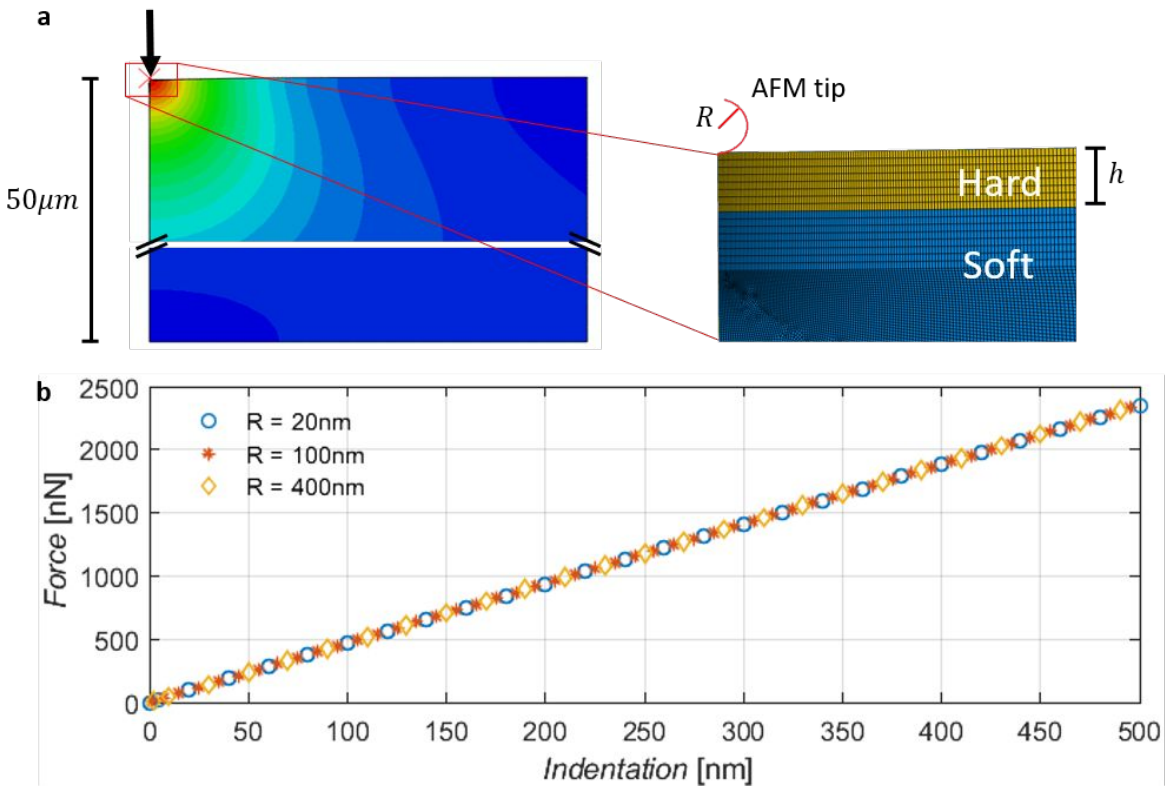

Figure S2. Finite Element Model. a) Example of vertical displacement map. Only the first 10 µm near the axis of symmetry are shown. The domain in the simulation is a square of side 50 µm. The inset shows details of the mesh in proximity to the indenter. b) Force-indentation curves obtained from numerical simulation with different indenter radii, namely 20, 100 and 400 nm.
